# Forecasted economic change and the self-fulfilling prophecy in economic decision-making

**DOI:** 10.1371/journal.pone.0174353

**Published:** 2017-03-23

**Authors:** Diamantis Petropoulos Petalas, Hein van Schie, Paul Hendriks Vettehen

**Affiliations:** Behavioural Science Institute, Radboud University, Nijmegen, the Netherlands; University of Pennsylvania, UNITED STATES

## Abstract

This study addresses the self-fulfilling prophecy effect, in the domain of economic decision-making. We present experimental data in support of the hypothesis that speculative forecasts of economic change can impact individuals’ economic decision behavior, prior to any realized changes. In a within-subjects experiment, participants (N = 40) played 180 trials in a Balloon Analogue Risk Talk (BART) in which they could make actual profit. Simple messages about possible (positive and negative) changes in outcome probabilities of future trials had significant effects on measures of risk taking (number of inflations) and actual profits in the game. These effects were enduring, even though no systematic changes in actual outcome probabilities took place following any of the messages. Risk taking also found to be reflected in reaction times revealing increasing reaction times with riskier decisions. Positive and negative economic forecasts affected reaction times slopes differently, with negative forecasts resulting in increased reaction time slopes as a function of risk. These findings suggest that forecasted positive or negative economic change can bias people’s mental model of the economy and reduce or stimulate risk taking. Possible implications for media-fulfilling prophecies in the domain of the economy are considered.

## Introduction

Greece, June 2015: The country’s economic situation forced capital controls prompting a three-week shut down of the banks. During this period, the majority of national and international news organizations issued speculative news about a possible withdrawal of Greece from the Eurozone (Grexit) and about the economic consequences of imminent capital control measures. The national news media further speculated on prospect blockages in people’s savings accounts and safe deposit boxes (see [1] as an example). Also during this period, individuals flocked daily in front of ATMs to withdraw the maximally allowed amount of cash from savings accounts. On July 20 when the banks opened again, customers thronged to access deposit boxes. The example may be explained by a self-fulfilling prophecy effect: a change in people’s mindset about future economic prospects affected their economic decisions in ways that potentially could contribute to making the economic prospects come true.

### Self-fulfilling prophecies in economic decision-making

A self-fulfilling prophecy occurs when the adoption of a belief affects behavior in such a way, that this belief becomes a reality. Self-fulfilling prophecy effects have been a topic of investigation in psychological research already from the 1960s, focusing mostly at the interpersonal level [2–4]. However, already in the 1940s, sociologist Robert Merton discussed implications of the self-fulfilling prophecy effect for the domain of the economy, in fact using a very similar example as in the opening paragraph [5]. Recently, studies in finance and communication research have presented empirical findings in line with the idea that media economic news are responsible for stimulating or attenuating effects in the larger economy [6–35]. For instance, studies using content analyses have identified different frames used in reporting of economic news in the media [20–23], and studies using sentiment analyses have found correlations between tone and/or sentiment of media economic news or macro-economic announcements and changes in leading economic indicators [6,16,24–31]. By and large, these findings suggest that self-fulfilling prophecies may very well apply in the domain of economic decision-making: News provides people with a general belief on the direction of the economy, which in turn influences economic choice.

Remarkably however, the idea that economic forecasts may influence people's economic decisions has not been investigated at the level of *individual* decision-making, as most studies in finance and communication science have either focused on market level responses to economic news or have measured individual responses to economic news at the level of attitudes and not actual economic behavior of individuals [8,14,32]. Note furthermore that studies in psychology do typically measure individual economic decision-making. However, these studies have mostly focused on the affective influences and emotional processes contributing to individual economic decision-making (reviews in [36,37]), for instance by manipulating framing [38], priming of emotions associated with market booms or burst [39], and emotional affect associated with winning and losing [40]. Instead, the current study is directed at understanding to what extent a cognitive belief or prediction about the future of the economy per se may impact economic decision-making and cause a self-fulfilling prophecy effect in one’s economic situation.

### The present study

Our study investigated the idea that simple messages forecasting changes in economic conditions can impact behavioral decision-making, even before any change has manifested. Such an effect would help to identify whether economic forecasts could affect an individual’s decision-making exhibiting a self-fulfilling prophecy effect. To simulate a realistic decision-making situation that resembles real-world economic choice, we used the Balloon Analogue Risk Task (BART) [41]. The BART can be seen as a visual metaphor of a bubble economy at risk of a burst, without knowing the exact point of burst.

In the original version of the BART, participants can acquire an increasing amount of money by inflating a visual analogue of a balloon up to a point considered optimal. At every successive inflation-step of the balloon, the accumulated value of the balloon increases, but so does the probability for the balloon to burst. Therefore, each inflation step comes at the risk of bursting the balloon and losing the value of the balloon accumulated up to that point. At any moment, players can choose to withdraw and stop inflating the balloon as to save the acquired amount to that point, and subsequently start inflating a new balloon.

Analogous to news forecasts that make predictions about the economy, we used simple predictive messages about the BART economy about a possible change in the size (smaller or larger) at which balloons may burst. Importantly, although the points of balloon bursts varied over trials in the game, the probabilities for balloon bursts associated with each inflation step were kept unchanged (and disclosed from participants) throughout the game. Crucially, because of the inherent uncertainty in the probability of balloons’ points of bursts any predictive message about the state of the BART economy would be very difficult to check for participants, analogous to news messages about the real economy.

The BART has been used previously to investigate framing effects [42–44] and effects of emotion on risky choice [45–49]. To the best of our knowledge, there are no earlier investigations of the self-fulfilling prophecy effect using the BART.

#### Hypotheses and expectations

The core theoretical idea that is investigated in this study is that forecasts about possible economic changes may influence people’s perception of risk and consequently their economic decision-making, even before any actual change has become apparent. This could provide a basis for a self-fulfilling prophecy effect in individual economic decision-making. If this is true, we should be able to see direct effects of economic forecasts on financial decision-making in the BART. Accordingly it was hypothesized that (H1) messages about possible negative (positive) economic changes will result in reduced (increased) risk taking. At the operational level, we expected the effect of forecasted economic change to be visible in the mean number of balloon inflations and consequentially the number of balloon bursts. We also expected this effect on risk taking responses to be consistent across time, as economic forecasts induce a change in perspective, and not a transient emotional response. Furthermore, in line with the self-fulfilling prophecy effect, we anticipated economic messages to affect participants’ actual earnings in the game.

Fundamental to changes in actual risk taking, we argue that economic forecasts may affect perception of risk. In the BART, risk perception can be measured implicitly through reaction times (RTs) [50–52]. The more risky a decision is, in terms of value at stake and probability of losing, the longer it should take participants to make a choice. Accordingly, we hypothesized that (H2) the increase in risk with each consecutive step in the BART will be accompanied by an increase in RT. More importantly however, we reasoned that any change in perceived risk following positive or negative messages about possible changes in future economic conditions in the BART should also become evident in reaction times. More specifically we hypothesized that (H3) the increased (decreased) perception of risk following negative (positive) forecasts should accompanied by a steeper (more flattened) increase in RTs over inflations. At the operational level we thus predicted that successively riskier choices (inflation steps) in the BART would result in increased RTs and most notably, that RTs would show larger increases in anticipation of negative changes, compared to positive.

## Materials and methods

### Design

Our study used a within-subject experimental design with three levels (blocks): a baseline and two experimental blocks. The baseline block was always presented first. Both experimental blocks were preceded by a message forecast that was either positive or negative. The order of the positive and negative messages was counterbalanced across participants. The study has fully complied with APA’s and with Declaration of Helsinki’s statements of ethical principles for psychological research involving human subjects. Participants provided both a written and active consent for taking part in the study. The Ethics Committee Faculty of Social Sciences (ECSS) at Radboud University, the institute where the research was conducted, has approved the study.

### Participants

To estimate the sample size necessary for the study we run a priori power analysis using G*power version 3.1.9.2. [53]. Assuming statistical power of β = .90, α-level of .05 and a conservative effect size *d* = .25 in a 3-block within-subjects design, we recruited a total sample of 40 university students (male = 17, female = 23), who were tested in the lab. Their age ranged from 18 to 33 years (*M* = 23.4; *SD =* 3.3 years). Each participant was offered a standard participation credit and additionally the chance of winning one out of five checks of 25.00 euro, based on their performance in the BART.

### Risk taking in the BART

**Fig 1:**
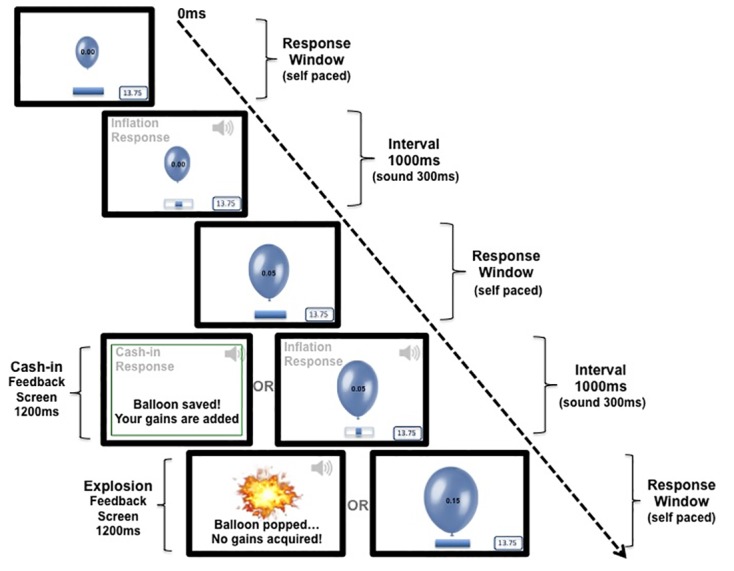
Time-course of events at a given trial of the BART task. Note: Every inflation response was followed by an interval of 1000ms during which the participant heard a pumping sound and saw a process bar loading. The response resulted in either an increase in the balloon size or to a balloon burst. Following a cash-in response or a balloon burst, positive and negative feedback screens (visual and auditory feedback) were presented respectively, for 1200ms.

As described, the BART is an economic decision game in which participants can make actual profit by taking successive risks. In the modified version of the BART we used in our study, each balloon could be maximally inflated 12 times before a definite burst occurred. At any moment, players could choose to withdraw and stop inflating the balloon as to save the acquired amount to that point, and subsequently start inflating a new balloon. This way the BART visually resembles a balloon economy and simulates realistic decision-making, as each balloon inflation is a risky decision (with unknown, yet quantifiable, first order risk probabilities over time) that can result either in an increase of possible gains or in a loss of the gains accumulated up to the point of burst for each balloon. See Fig 1 for a schematic illustration of the BART.

For creating the experiment we used *Presentation* version 15.0 software (Neurobiological Systems inc., USA, San Francisco Bay Area). A gaming keyboard was used for collecting responses to maximize temporal accuracy of RT recordings; the space bar was used for inflate responses and the left control button was used for collect (cash-in) responses. Participants were instructed to use their right hand for inflating the balloon and their left hand for cashing-in. Prior to the task participants were only told that the balloon could be inflated up to a maximum of 12 times and that a certain explosion would occur at that point. Participants were further instructed to inflate at will until an explosion occurred or before deciding to cash-in; subsequently they would start inflating a new balloon. At the beginning of each trial, participants viewed a picture of an oval blue balloon (initial dimensions: 3 cm high x 2 cm twelve: 36 cm high x 24 cm wide) at the midpoint of a 21” computer screen (white background) with the monetary value set to 0.00 in its center (font type: Arial; font size: 12 pt.), a box displaying the gains earned at the bottom right of the screen, and a blue indeterminate process bar was displayed below the balloon picture. Each inflation response was followed by a fixed interval of 1000ms, during which an inflation sound (300ms) was played and the process bar loaded for 1000ms. After this interval the balloon either increased in size (discretely) by 8%, or exploded. In case of a successful inflation, the wager value at the center of the updated balloon picture increased (see Table 1 for a detailed description of the successive wagers at each inflation step). In case of an explosion a negative feedback screen was presented on the screen for 1200ms, during which a picture of an explosion, an explosion sound (900ms) and the text “Balloon popped… No gains acquired” were displayed. A cash-in response resulted in presentation of a positive feedback screen for 1200ms, during which a clinking sound (200ms) was played and the text “Balloon saved! Your gains are added” *was displayed*. After presentation of either the positive or the negative feedback screen, participants could start inflating a new balloon.

**Table 1:** Probabilistic and pay-off structure in the BART Task.

Number of inflation 1–12	Gamble associated with each inflation	Probability (*p*) of burst for each inflation step
1	0.05	0.0
2	0.15	0.15
3	0.25	0.23
4	0.40	0.30
5	0.60	0.38
6	0.85	0.46
7	1.15	0.53
8	1.50	0.61
9	1.90	0.69
10	2.35	0.76
11	2.85	0.84
12	3.20	1

*Note*. In our study, we used a maximum point of 12 inflations per balloon (trial), with a 0.0% probability of a burst for the first inflation increasing up to 100% in inflation number 12.

### Procedure

Participants were familiarized with the BART by first playing 5 practice trials (balloons). Subsequently, participants were presented with a baseline block of 60 trials and with two more blocks of 60 trials each. At the end of each block of 60 trials, a text message (font type: Arial; font size: 28; font color: navy blue) on a black background appeared on the screen, informing participants they could rest for maximum three minutes (self-paced) before they began with the next set of balloons. Before each of these two experimental blocks participants were presented with either a positive or a negative message–counterbalanced–on a black background (font type: Arial; font size: 36; font color: ochre). These messages only introduced a *possibility* that an economic change might occur at some point, were neutrally framed and only differed in valence. Specifically, the messages read as follows: “Positive (negative) economic changes may occur within the coming trials. In this case, the chances of explosions will decrease (increase), which will result in balloons popping at bigger (smaller) sizes, and can influence your total gains”.

Following all 180 trials of the BART, participants completed six open questions and 18 scale items on a seven-point Likert scale (1 = not at all, 4 = average, 7 = completely) measuring different aspects of their experience of the game, including items about trust derived from the messages (“Did the messages you received influence your expectations on the trials that followed?”), change of strategy (“Did you change your decision strategy after the positive/negative message was presented?”), change in attention (“I was paying more attention in the game, after the positive/negative message was presented”), actual change noticed (“Did you notice changes in the balloons’ points of burst following presentation of the positive/negative message?”), and affect resulting from each of the two messages (“I felt more relaxed/anxious while playing the game, in the trials that followed the positive/negative message”); results involving these variables are reported below. The questionnaire further included 15 closed questions (socioeconomic status, age, gender, education level) and 20 scale items adopted from the DOSPERT scale [54], in order to check for individual differences in impulse-related behaviors (e.g. gambling, use of medicine, recreational substances, tobacco and alcohol); none of these variables were found to relate with task performance. The experiment lasted 45–55 minutes, based on participants’ pace.

### Data analysis

To test our first hypothesis we calculated risk taking as the average number of inflations per balloon for those trials in which the balloon did not burst separately for each block. Following convention, we also calculated and report number of balloon bursts per block per participant. We then compared differences in these scores between the three blocks using repeated measures analysis of variance (ANOVA).

To address our second and third hypotheses, we recorded RT data on inflation responses and we calculated averaged RTs as a function of inflation steps across all trials. We then tested for differences in RTs as a function of inflation steps by means of a repeated measures ANOVA, and we used regression analysis techniques and a simple t-test to compare the difference in the steepness of the RTs function slopes between the two main experimental blocks. All data were preprocessed and analyzed using IBM SPSS Statistics for Macintosh (version 20.0, Armonk, NY: IBM Corp). All data were analyzed anonymously.

## Results

### Behavioral performance in the BART

The analysis of risk taking was limited to balloons for which the subjects chose to stop so that no explosion occurred. In all three blocks, the number of “balloons collected” (instances in which participants opted for collecting gains and thus to stop inflating the balloon) was greater than the number of “balloons bursts” (instances in which the balloon exploded)–see Table 2 for descriptive statistics.

**Table 2:** Behavioral Performance in the BART Task: Descriptive Statistics.

Block	Variables	*N*	Min	Max	*M* (*SD*)	*SE*	*95% CI*
Baseline (no message)	Nr of adj. inflations	40	3.69	8.25	5.85 (1.03)	.163	[5.52, 6.19]
	Balloon bursts	40	12	36	25.20 (5.52)	.873	[23.43, 26.96]
	Balloons collected	40	24	48	34.80 (5.52)	.873	[33.03, 36.56]
	Actual earnings	40	19.65	44.55	32.30 (5.95)	.885	[30.51, 34.08]
Negative message	Nr of adj. inflations	40	2.85	8.04	5.29 (1.09)	.173	[4.95, 5.65]
	Balloon bursts	40	11	36	22.00 (6.87)	1.087	[19.80, 24.19]
	Balloons collected	40	24	49	38.00 (6.87)	1.087	[35.80, 40.19]
	Actual earnings	40	15.75	41.25	29.84 (5.85)	.926	[27.97, 31.71]
Positive message	Nr of adj. inflations	40	4.27	8.95	6.24 (1.13)	.179	[5.88, 6.60]
	Balloon bursts	40	15	40	27.85 (6.70)	1.059	[25.70, 29.99]
	Balloons collected	40	20	45	32.15 (6.70)	1.059	[30.01, 34.29]
	Actual earnings	40	22.85	45.25	32.09 (5.12)	.810	[30.45, 33.73]

*Note*. Number of adjusted inflations refers to the average number of inflations in trials for which the balloon did not burst.

#### Analysis of balloon inflations, balloon bursts and actual earnings

Some subjects inflated the balloons as many as eleven times. Inspection of the raw data revealed that only four single trials from one corresponding subject scored three or more standard deviation points above the mean. In the analysis we removed these data points. The findings reported have not been influenced by this choice.

All data were normally distributed, as assessed by boxplot and Shapiro-Wilk test (*p* > .05). We first ran a 3 x 2 repeated measures ANOVA to test for order effects (Order as a between subject variable). The analysis showed that neither the effect of Order, *F*(1 38) = .279 *p* = .601; partial η^2^ = .007, nor the interaction between Order and Block (*F*(2 76) = .848, *p* = .433, partial η^2^ = .022) were significant. Thus, we proceeded by assuming one homogenous group of 40 participants, and tested our first (H1) main hypothesis using one-way repeated measures ANOVA tests with three levels (Block: baseline, negative, positive). The assumption of sphericity was met, as assessed by Mauchly’s test of sphericity (χ^2^(2) *=* .970 *p* = .565).

**Fig 2:**
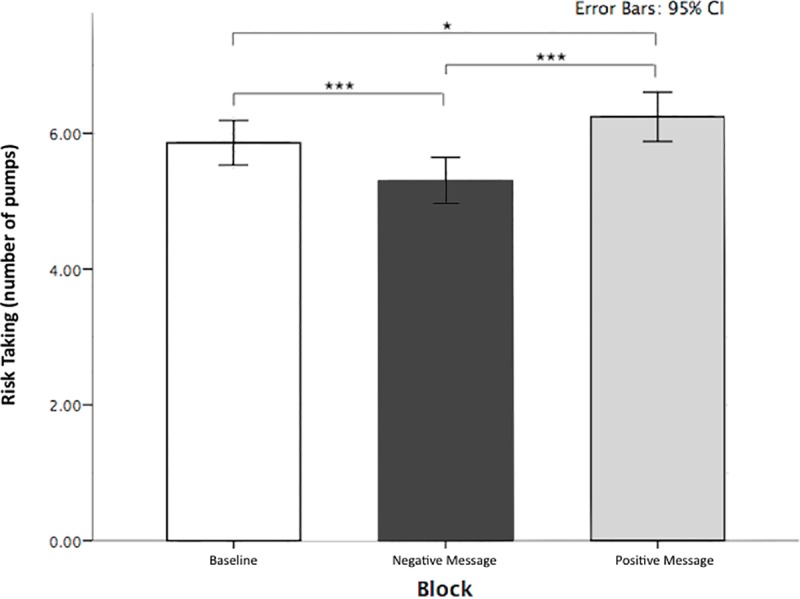
Risk taking in the BART task as a function of number of inflations in each experimental block. Note: The dashed line represents performance in the baseline; error bars show 95% confidence intervals; asterisks indicate significance values (*p < .05, **p < .005, ***p < .001).

The analysis showed a main effect of Block *F*(2,78) = 26.446, *p* < .001, partial η^2^ = .404. In support of our first (H1) hypothesis, planned contrasts showed that compared to balloon inflations at baseline (*M* = 5.86, *SD* = 1.03) participants inflated the balloon significantly less strongly (less risk taking) following the negative message (*M* = 5.29, *SD* = 1.09), an effect of *F*(1,39) = 18.733, *p* < .001, partial η^2^ = .324; and they inflated more (more risk taking) after the positive message (*M* = 6.24, *SD* = 1.13), an effect of *F*(1,39) = 7.405, *p* = .010, partial η^2^ = .160. The difference in inflation scores between the negative and the positive message was significant, *F*(1,39) = 34.001, *p* < .001, partial η^2^ = .466. See Fig 2 for a schematic illustration of these results.

To (post-hoc) explore the idea that the negative (compared to the positive) anticipation could result in a stronger prediction bias, we computed difference scores for inflation responses between the negative forecast block and the baseline (*ΔM* = .57, *r* = .569, *SE =* .130, 95% *CI* [0.24, 0.89]) and between the positive forecast block and the baseline (*ΔM* = .38, *r* = .399, *SE* = .141, 95% *CI* [0.31, 0.73]). Subsequently, we performed a post hoc paired t-test to compare these differences and found that the negative message showed a larger difference from the baseline, compared to the positive message (*t*(1,39) = 7.811 *p* < .001 *d* = .211), in support of the idea of a stronger prediction bias following negative messages than positive messages.

Following convention, we ran similar analyses for testing differences in balloon bursts. There were no outliers and data were normally distributed in all three blocks, as assessed with boxplots and the Shapiro-Wilk test (all *p* values were > .05), respectively. The assumption of sphericity was met (Mauchly’s test: χ^2^(2) = 4.807, *p* = .090). As expected, differences in balloon bursts were found statistically significant (main effect of Block) across the three blocks of trials *F*(2, 78) = 22.381, *p* < .001, partial η^2^ = .365. Planned contrasts showed that compared to balloon bursts in the baseline (*M* = 25.20, *SD* = 5.52) participants burst less balloons following the negative message block (*M* = 22.00, *SD* = 6.87), an effect of *F*(1, 39) = 9.932, *p* = .003, partial η^2^ = .203, while they burst more balloons following the positive message block (*M* = 27.85, *SD* = 6.70), an effect of *F*(1, 39) = 10.965, *p* = .002, partial η^2^ = .219. Similar to the analysis of the number of inflations, we (post-hoc) explored differences in change scores for balloon bursts between the negative forecast block and the baseline and between the positive forecast block and the baseline. Following the negative message, the change in balloon bursts from the baseline was larger compared to the positive message, and a post-hoc paired samples t-test showed that this difference was significant (*t*(1,39) = 7.733 *p* < .001 *d* = .113).

Significant differences were also found in actual earnings made in the BART across the three blocks: one-way repeated measures ANOVA with three levels (baseline, negative message condition, positive message condition) revealed a main effect of Block, *F*(2, 78) = 3.805, *p* = .026, partial η^2^ = .089. On average, earnings made in the negative message condition (*M* = 29.84 €, *SD* = 5.85, *SE* = .926, 95% *CI* [27.97 31.71]) were significantly lower compared to earnings made in the baseline (*M* = 32.30 €, *SD* = 5.95, *SE* = .885, 95% *CI* [30.51 34.08], *p* < .01), as well as earnings made in the positive message condition (*M* = 32.09 €, *SD* = 5.12, *SE* = .810, 95% *CI* [30.45 33.73], *p* < .05); there was no significant difference between the positive and the baseline conditions (*p* = .843).

#### Stability of the effect over time

To test how persistent the influence of economic forecasts may be, we further tested whether the observed effect of the messages on risk taking was stable across the sequence of all trials of each block. the messages induced a persistent change in the perception of costs and benefits associated with decisions in the task, one would expect the effect to be consistent throughout each of the two experimental blocks. This would provide an indication for a more general shift in participants’ mental model of the BART economy, rather than a transient effect of arousal or emotional priming.

**Fig 3:**
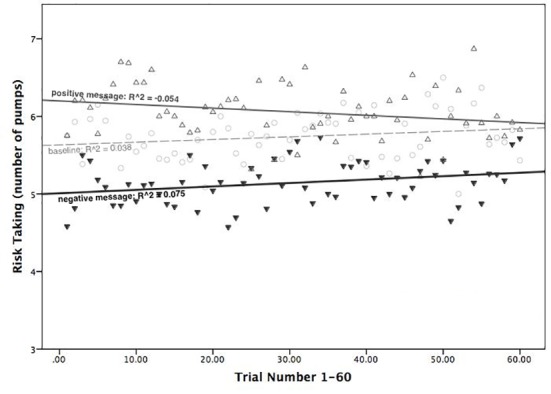
Risk taking as a function of trial number 1–60, for each block of trials. Note: middle dashed line and circles indicate performance in the baseline; bottom bold black line and down-pointing arrows denote performance in the negative message block; top solid grey line and up-pointing arrows denote performance in the positive message block. No significant changes in risk taking across 60 trials were found in the two experimental blocks.

To statistically test this prediction, we compared the effect of the messages on the number of inflations in the first half (30 trials) versus the second half (30 trials) of all trials in each block. We used a 3 x 2 within subjects ANOVA, with three levels for the factor Block and two levels for the factor Time (Time1: first half of 30 versus Time 2: second half of 30 trials). Neither the effect of Time *F*(1, 76) = .172 *p* = .680 partial η^2^ *=* .002 nor the Block x Time interaction *F*(1.64, 125.08) = .510 *p* = .602 partial η^2^ = .007) was significant. To visually illustrate performance over time, we also regressed mean number of inflations on trial number in each block (see Fig 3). We found no significant change in risk taking over the course of 60 trials for none of the two experimental conditions (*R*^*2*^_*negative*_ = .075, β_negative_ = .073; *R*^*2*^_*positive*_ = −.054 β_positive_ = -.006).

### Reaction times (RTs) in the BART

To test our second (H2) and third (H3) hypotheses, we computed average RTs associated with the decision to inflate across all participants and for each of the two experimental blocks. Because the first inflation step was associated with a zero probability for the balloons to explode and because reaction times were always slower compared to the subsequent steps reflecting the change of screen to the new trial, it was removed from the analysis. Visual inspection of RT data using histograms and boxplots indicated that responses above 3000 milliseconds represented outliers. We calculated *z* scores of RT data for each participant per block and inflation step, and removed data points that scored three or more standard deviation points above the mean for the corresponding observations. The removal of these data resulted in reducing the total number of cases by 2.8%, without affecting the results reported below. For an overview of the descriptive statistics for RT data, see Table 3.

**Table 3:** Descriptive Statistics for Reaction Times (RT) in the BART Task.

Number of Inflation Step	Block	*n (SD)*	*Mean RT* (*SD*)	*SE*
Step 2	Baseline (no message)	59 (1.14)	308.71 (358.12)	7.44
	Negative Message	59 (1.60)	225.22 (260.81)	5.52
	Positive Message	59 (.80)	255.62 (343.87)	7.29
Step 3	Baseline	55 (2.95)	319.33 (358.70)	7.71
	Negative Message	53 (4.58)	228.03 (297.41)	6.59
	Positive Message	54 (2.46)	249.26 (347.53)	7.65
Step 4	Baseline	48 (4.83)	353.34 (371.98)	8.57
	Negative Message	45 (9.86)	245.35 (297.11)	7.14
	Positive Message	48 (4.60)	256.39 (349.21)	8.16
Step 5	Baseline	39 (9.68)	432.16 (456.91)	11.60
	Negative Message	35 (10.98)	276.90 (338.55)	9.33
	Positive Message	41 (7.74)	276.10 (349.98)	8.87
Step 6	Baseline	29 (12.23)	465.42 (442.99)	13.26
	Negative Message	23 (12.39)	307.99 (322.81)	11.13
	Positive Message	31 (9.28)	294.12 (337.01)	9.99
Step 7	Baseline	17 (10.14)	551.58 (516.02)	20.60
	Negative Message	13 (10.10)	339.96 (342.70)	16.26
	Positive Message	20 (10.91)	313.87 (326.19)	11.97
Step 8	Baseline	7 (6.58)	598.92 (473.57)	31.09
	Negative Message	6 (5.83)	446.99 (432.17)	35.40
	Positive Message	10 (8.07)	380.04 (392.52)	21.04

*Note*: *n* refers to the mean number of trials preserved for the analysis of RTs, per subject; in parenthesis, the standard deviation. *Note*. The first step (inflation step number 1) was excluded from all analyses (see text).

#### Increase of RTs as a function of riskiness of the decision

Each successive inflation response in the BART is a decision associated with a higher level of perceived risk. Hence we predicted RTs to increase as a function of inflation step. Furthermore, we expected this upward slope in RTs to increase more steeply following the negative (compared to positive) economic, as an implicit measure of participants’ biased perception of the BART reward variance. We computed average RTs per inflation step for each participant and we used a criterion of at least ten cases per data point. We obtained data for all participants (*N* = 40) up to inflation step number six, as many participants did not take as much risk as more than six inflations. Therefore, we then ran a 5 x 2 repeated measures ANOVA with *Step* (inflation step 2–6) and message Block (positive vs. negative) both as within subject factors, to test for RT differences between the two experimental message blocks. As Mauchly’s test of sphericity indicated that the assumption of sphericity had been violated, χ^2^(9) = 33.842 *p* <.001, Greenhouse-Geisser corrected tests are reported (ε = .68). We did not find a significant main effect of Block (*p* = .675), however we found a strong effect of Step *F* (1.829, 71.348) = 36.221, *p* < .001, partial η^2^ = .482 and a strong interaction (Step x Block) effect *F* (2.738, 106.789) = 7.737, *p* < .001, partial η^2^ = .166, indicating that the RTs change as a function of the increase in inflation step was different between the two experimental blocks.

**Fig 4:**
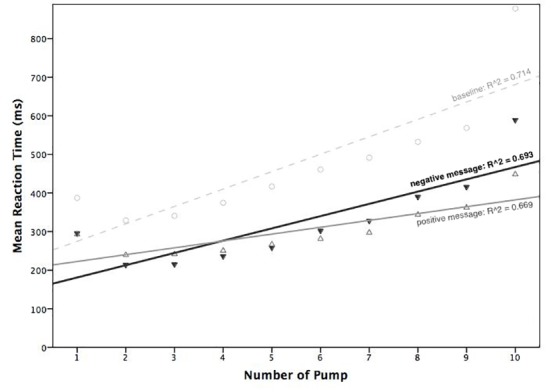
Averaged regression lines of reaction times (RTs) for inflation responses, as a function of increase in inflation step number, for each of the three blocks of trials. Note: the top light grey dashed line represents averaged regression slope in the baseline. The two bottom dark solid and grey dashed lines show averaged regression slope in the negative and in the positive message blocks, respectively. The first inflation step was excluded from all analyses (see text).

Inspection of averaged RTs per condition across inflation steps (see Fig 4) suggested that the slope of RTs across step was steeper in the negative than in the positive forecast message block. An additional analysis was therefore conducted to investigate the difference in steepness of the RT slopes in the two main experimental blocks; to include more data points in the analysis we included inflation steps beyond number six. Using linear regression, we first regressed average RT scores on inflation step number, separately for each participant and for each of the two main experimental blocks. The regression analysis accounted for approximately 85% of the variance in RTs for the negative (*R*^2^_negative_ = .693) and the positive (*R*^*2*^_positive_ = .669) message blocks, reflecting the reliability of this approach. We then extracted the β values and intercept values for each of the two experimental blocks, and eventually compared how differently RTs changed as a function of the increase in inflation step between the two experimental blocks. A paired-samples *t*-test was finally used to statistically test the difference in β values, i.e. the mean steepness of the two averaged regression slopes. The assumption of normality was not violated, as assessed by Shapiro-Wilk’s test (*p* = .145). We found that the negative economic message elicited a stronger increase in the rate of change of RTs per inflation step compared to the positive message, *t*(39) = 2.514 *p* = .016 *d* = .397.

### Subjective experience in the BART

Besides the behavioral measures reported so far we also obtained self-reports that may provide some further information on the way that participants’ experienced the game and their trust in the messages that were presented. Participants’ answers on the seven-point Likert scale (1 = not at all, 4 = average, 7 = completely) indicated that they experienced some anxiety in response to the negative message (*M* = 3.58, *SD* = 1.43, one-sample t-test χ^2^(39) = 15.809, *p* < .001) and were somewhat relieved in response to the positive message (*M* = 3.78, *SD* = 1.42, one-sample t-test χ^2^ (39) = 16.778, *p* < .001). They reported that both messages (positive and negative together) had influenced their expectations in the trials that followed (*M* = 5.10, *SD* = 1.72).

To explore possible differences between the (positive and negative) message conditions we ran paired t-tests on the corresponding self-reports. Participants reported that they changed their strategy after the positive (*M* = 4.93, *SD* = 1.65) and after the negative (*M* = 4.63, *SD* = 1.90) messages were presented, yet this difference was insignificant (*p* = .142). Participants reported to have been more attentive following presentation of the negative (*M* = 3.93, *SD* = 1.97) message than following presentation of the positive message (*M* = 3.33, *SD* = 1.40), *t*(39) = 2.726, *p* = .010). However, they did not report being more or less careful in their decisions after receiving the negative (*M* = 4.03, *SD* = 1.76) than following the positive (*M* = 4.35, *SD* = 1.68) message (*p* = .465). Lastly, participants reported that they noticed a stronger change in the game following the positive (*M* = 4.88, *SD* = 1.66) than following the negative (*M* = 3.45, *SD* = 1.89) message, *t*(39) = 2.903, *p* = .006.

## Discussion

In this study, we used the BART to test the hypothesis that economic forecasts can bias people’s beliefs of the economy and influence their economic decision-making. Such a mechanism would contribute to the discussion about self-fulfilling prophecy effects in economic decision-making whereby the belief that economic change may come about will cause people to act as if as this reality has already set in, causing the anticipated change to become an actual reality. In support of this hypothesis, our study showed that speculative messages forecasting a possible change in economic conditions influenced recipients’ beliefs about the riskiness of their choices (as reflected in RTs) and affected the riskiness of their economic decision-making (number of balloon inflations) over a series of 60 trials in the BART. These findings are in accord with the idea that economic forecasts may unwillingly influence economic decisions such that the forecasted economic change becomes a reality.

In line with the proposal that economic forecasts in media news may generate a self-fulfilling prophecy effect [6,8,11–17,19,25–29,31,33–35] positive and negative economic messages caused participants to take more and less risk in the BART, respectively (Fig 2). Participants acted in accordance with the forecasted reality of the BART economy, adapting their risk taking as if the predicted change had already occurred, whereas in fact no change had occurred at all. We suggest that in conditions of strong ambiguity (e.g. it is unclear whether the economy is going up or down) and when data may be consistent with either perspective (i.e. examples of positive and negative economic development can found in both rising and declining economies) people have little opportunity to detect and correct false forecasts and beliefs through sampling. Although no changes occurred in balloons’ probabilities of bursts, following either the positive or negative message, participants did not pick up on this lack of change. Furthermore, we found that the effect of positive and negative messages on risk taking was stable over time (Fig 3). This suggests that the adopted economic belief generated a continuous influence on risk taking throughout the block of 60 trials. Not surprisingly, participants made less money in the negative message condition than in the positive message condition. Hence a false prediction about a negative or a positive change in the BART economy was accompanied by an actual decrease or increase in participants’ earnings, exhibiting the self-fulfilling prophecy effect.

In accordance with our second hypothesis, we found that reaction times for inflation responses increased as the risk associated with each successive inflation step increased, in all three blocks. This general increase in reaction time reflects more time for processing potential risks and benefits associated with successive decisions in the BART: the more risky the decision became, in terms of stake value and chance of winning or losing, the longer it took participants to make a decision [50,51]. Reaction times in the baseline block were substantially higher compared to the two experimental blocks. This may reflect a high initial uncertainty about the implicit pay-off model underlying the BART and/or extra time associated with learning a new task, considering that the baseline was always presented first. Most notably and in line with our third hypothesis, the rate of increase in RTs was greater for the negative than for the positive message condition (Fig 4). This finding in RTs supports our idea that the effect of economic forecasting on risk taking (i.e. the number of inflations) reflects a change in perceived riskiness of successive inflation steps in the BART task. That is, participants appeared to have adjusted their mental model of the BART economy following the forecast of economic change.

### Predictive coding of forecasted economic change

Recent advances in computational modeling and cognitive neuroscience have presented a unifying framework for brain functioning and human cognition [55–60] that may apply particularly well in explaining how beliefs about the economy may direct economic decision-making. According to the predictive coding framework, brains are essentially hierarchical prediction machines that try to match higher-level hypotheses (e.g. beliefs about the state of the world) with lower-level (perceptual and conceptual) input [55]. People’s view about the state of the economy may be considered a typically high level belief that is shaped by accumulating evidence at lower levels (e.g. information about different economic indicators as well as more individual experiences). Priors high up in the hierarchy are general beliefs that are usually quite stable over time and that do not generate very precise predictions at lower levels. Consequently, it will take a considerable amount of time and evidence for people’s beliefs about the economy to change gradually through sampling. However, general economic forecasts may more directly and immediately influence public opinions about the economy [61], for instance by presenting conclusions that directly address prior beliefs (as for instance in the headline ‘Here’s one more sign the US economy is slowing’ [62]). As indicated earlier, such conclusions are difficult to falsify considering the ambiguous nature of economic indices. Hence, when a false belief is adopted, it may be difficult to change through repeated sampling over time. Consequently, incorrect biases in people’s belief about the economy may continue to influence economic decision-making, until another convincing economic perspective is presented that biases belief in a different direction. In line with this idea, visualization and regression of risk taking (nr. of inflations) as a function of trial number (Fig 4) indicated that effects of positive and negative economic forecasts set in quickly and remained stable over the course of the block. Furthermore findings in RTs, and specifically the increase and decrease in RT slopes with step size following negative and positive economic messages, corroborate the idea that the economic messages effectuated a change in participants’ psychological reality, i.e. the perception of more or less risk of popping the balloon. These findings are consistent with a predictive coding account wherein changes in belief bias people’s perception and behavior.

### Affect as an alternative explanation

The current study investigated to what extend forecasted economic change may influence cognitive economic decision-making of individuals. A number of previous studies using either the BART [45–49] or other behavioral measures [63–66] have shown that manipulation of emotional affect may also bias economic risk taking. Affective reactions can be particularly salient in circumventing uncertainty and expediting the decision-making process [67]. Consequently, emotional affect may present an alternative explanation for our study’s findings. Although affective states undoubtedly played a role in the current experiment, as for instance reflected in self-reported levels of relief and anxiety following messages of positive and negative economic forecast, there are several arguments that go against the idea of affective states as the major explanation of the findings. First, although participants indicated that they had experienced some relief and anxiety in response to positive and negative massages, ratings showed that these self-reported affective responses were moderate at most and below the average of the scale (<4). Instead, participants were more affirmative with respect to the question if messages had influenced their expectations in the trials that followed (>5) which is in line with the fact that message text were constructed to bias participants expectations, and not to trigger affect. Second, and more convincingly, we found that positive and negative messages exerted a continuous effect on economic risk taking throughout the block, across a series of 60 trials. This pattern of results is incompatible with an affective explanation, as one would expect the effect to dissipate as the initial emotional response disappears and the participant adjusts to the new conditions [45,46]. We note here that a predictive coding account would also predict a gradual correction of incorrect beliefs over time. Considering the noisy and unpredictable nature of the BART, however, such a correction would likely require many more trials than the limited number of 60 trials per block that was included in the present experiment. In all, we argue that our manipulation of economic forecast influenced participants’ decision-making mainly through cognitive means, that is, by changing participants’ beliefs about the BART economy.

### Limitations

Obviously, making decisions in the lab where only small amounts of money can be gained differs from making economic decisions in real-life, were the stakes are much larger. In addition to this, participants in this study could either win or not win, but never loose money. Consequently, in the current study we only addressed behavioral performance in the gains domain, and not in the losses domain. However, we think that the overall pattern of the results observed here would be similar for larger stakes and possibly even amplified in the losses domain, similar to what has been found in earlier studies that investigated framing effects in gains and losses using the BART task [42–44].

The current study used a rather homogenous population, namely students, and differences in both behavioral and neural responsiveness between younger and older adults are well documented in existing decision-making literature [68–71]. However, we do think that the findings in our experiment capture the psychological reality of forecasted economic changes on decision-making that applies to both young and old. It would be interesting to investigate if the self-fulfilling prophecy effect manifests differently in younger and in older populations. However, we would be surprised if the general effect of economic forecasts on decision-making would not be present in older adults.

Last, in our study post-hoc calculation of effect sizes suggested that the observed effect of the messages on the number of inflations as a direct measure of risk taking was larger for the case of the negative (η^2^ = .324) as compared to the positive forecasts (η^2^ = .159). However, the baseline was always presented first and may not represent a neutral contrast. Therefore this effect deserves replication, using sufficient sample and a fully counterbalanced design. We note however that this pattern accords well with findings in other areas of research that have shown negative economic news to produce stronger responses in various indicators of economic activity, compared to positive [6,9,10,13,14,16,19,29,31,33–35].

### Broader implications

In addition to studies in economic psychology that have identified the important role of emotions in economic decision-making [45–49,63–67] the present study suggest that forecasted economic change may bias people’s psychological models about the economy and can induce a self-fulfilling prophecy effect. The idea that false beliefs can exert a strong influence on behavior could also extend to decision-making domains beyond the economy. Comparable effects may be found in the domain of politics, specifically in the case of elections. In predicting the results of an election, journalists may indirectly and unwillingly influence the outcome of these elections. Consistent with this idea Ansolabehere and Iyengar (1994) found that television newscast of election polls significantly biased voters preference towards the leading candidate who was forecasted to win, causing in a self-fulfilled prophecy [72]. Similar to the forecasts about economy, predictions of election outcomes are difficult to falsify and may consequently exert a strong bias on people’s beliefs and their subsequent behavior. It is important to note however that self-fulfilled prophecy effects are by no means limited to economic and political forecasting. Such effects may be witnessed in any system that is sufficiently complex, variable and noisy to the extent that claims or conclusions are difficult to falsify, such as e.g. in cases of religion and health care [73–75].

## Conclusion

Our study points out the importance of cognitive beliefs in economic decision-making in addition to emotional mechanisms that are known to drive people towards taking or avoiding financial risks. Forecasted economic change may cause immediate adaptations in people’s mental models of the economy which may be difficult to correct and that may influence the perception and sampling of economic information over a prolonged period of time. Furthermore, and importantly, incorrect beliefs about upcoming changes in the economy may bias economic decision-making such that forecasted economic change becomes a self-fulfilling prophecy.
